# Exploring Factors Affecting Health Care Providers' Behaviors for Maintaining Continuity of Care in Kerala, India; A Qualitative Analysis Using the Theoretical Domains Framework

**DOI:** 10.3389/fpubh.2022.891103

**Published:** 2022-07-08

**Authors:** Linju Joseph, Sheila Greenfield, Anna Lavis, T. R. Lekha, Jeemon Panniyammakal, Semira Manaseki-Holland

**Affiliations:** ^1^Institute of Applied Health Research, University of Birmingham, Birmingham, United Kingdom; ^2^Achutha Menon Centre for Health Science Studies, Sree Chitra Tirunal Institute for Medical Sciences and Technology, Thiruvananthapuram, India

**Keywords:** informational continuity of care, patient-held health records, health care provider perspective, theoretical domains framework, quality of care

## Abstract

**Introduction:**

Access to patients' documented medical information is necessary for building the informational continuity across different healthcare providers (HCP), particularly for patients with non-communicable diseases (NCD). Patient-held health records (PHR) such as NCD notebooks have important documented medical information, which can contribute to informational continuity in the outpatient settings for patients with diabetes and hypertension in Kerala. We aimed to use the theoretical domains framework (TDF) to identify the perceived HCP factors influencing informational and management continuity for patients with diabetes and hypertension.

**Methods:**

We re-analyzed semi-structured interview data for 17 HCPs with experience in the NCD programme in public health facilities in Kerala from a previous study, using the TDF. The previous study explored patients, carers and HCPs experiences using PHRs such as NCD notebooks in the management of diabetes and hypertension. Interview transcripts were deductively coded based on a coding framework based on the 14 domains of TDF. Specific beliefs were generated from the data grouped into the domains.

**Results:**

Data were coded into the 14 domains of TDF and generated 33 specific beliefs regarding maintaining informational and management continuity of care. Seven domains were judged to be acting as facilitators for recording in PHRs and maintaining continuity. The two domains “memory, attention and decision process” and “environmental context and resources” depicted the barriers identified by HCPs for informational continuity of care.

**Conclusion:**

In this exploration of recording and communicating patients' medical information in PHRs for patients with diabetes and hypertension, HCPs attributions of sub-optimal recording were used to identify domains that may be targeted for further development of supporting intervention. Overall, nine domains were likely to impact the barriers and facilitators for HCPs in recording in PHRs and communicating; subsequently maintaining informational and management continuity of care. This study showed that many underlying beliefs regarding informational continuity of care were based on HCPs' experiences with patient behaviors. Further research is needed for developing the content and appropriate support interventions for using PHRs to maintain informational continuity.

## Introduction

Continuity of care is essential to provide quality care to patients with diabetes and hypertension (1). Continuity of care refers to the seamless care over time between care episodes and across health care settings (2). Informational continuity is an essential component of continuity of care. Informational continuity refers to how efficiently patients' health information can travel with them across time, care episodes, and health care facilities (3, 4). Patients' health information can includes the patients' symptoms, treatment, management plan, test results, and other relevant details that are usually recorded (5). Having patient-level electronic or paper-based health records, which can be shared with all involved health care providers (HCP), is ideal for developing informational continuity (6). However, most low and middle income countries (LMICs) such as India have little or no patient-level paper-based or electronic health records available in the public health system (7). Health care in India is provided by public and private healthcare facilities. Patients tend to self-refer themselves to several doctors for their care for their condition/conditions. Therefore, in most cases patients/carers need to act as carriers of medical information and communicate with HCPs to ensure continuity of care. Without access to clearly documented and accessible health information regarding the patient, HCPs cannot provide safe and quality care over time. Furthermore, without past medical information or a shared management plan, medical management continuity for patients with diabetes and hypertension is hindered (3, 8). Due to the long-term nature of diabetes and hypertension, the nature of care needs is also varied over time, and hence information transfer and handover communication across visits and providers becomes essential (9). For example, with time a patient on oral diabetic medicine may need to change to insulin injection to maintain his/her blood glucose under control. The information needed for a patient and HCP when using oral medication and when on insulin injection are different. Factors hindering continuity of care include lack of readily available facility-based records, HCPs not recording the information, poor retention of verbal communication between HCPs and patients/carers, patients not being able to communicate essential details (7).

HCPs working in public health facilities in India are often overburdened with the workload (10). However, very few studies are conducted from the HCPs' perspective on current challenges for managing care for patients with diabetes and hypertension from Kerala (11, 12). Kerala is a south Indian state with a high burden of non-communicable diseases (NCD) particularly diabetes and hypertension (13). Patient-held health records have been recommended as a part of NPCDCS [National Programme for Prevention and Control of Cancer, Diabetes, Cardiovascular Diseases and Stroke (14)] to record and monitor health status of patients with diabetes and hypertension (15). The government of Kerala under the Aardram Mission aimed to transform existing primary care health centers to focal point of primary care by adding various services for patients with NCDs. HCPs can potentially use current patient-held health records for communication and maintaining continuity of care in the outpatient settings of public health settings in Kerala. However, based on a previous clinical handover study done in 2014, Himachal Pradesh and Kerala, HCPs' documentation in PHRs is sub-optimal (7). We explored the experiences of HCPs with PHRs in public health settings in Kerala using semi-structured interviews, as a part of larger qualitative study with patients and carers (under review in a journal, unpublished, own work). We found generalized awareness regarding the need for past medical information to manage patients with diabetes and hypertension. The recording in PHRs were sub-optimal and HCPs identified difficulties in locating information from multiple PHRs with patients. Therefore, to assist the development of appropriate supporting interventions to improve informational continuity of care, applying theory may offer new insights (16). The Theoretical Domains Framework (TDF) is an integrative framework that synthesizes many behavior change theories, which can help explain issues relating to the implementation of best practice evidence in healthcare settings (17). The TDF helps combine and simplify data and theories relating to a specific behavior into a set of theoretical domains (17). The revised version of the TDF contains 14 theoretical domains (Table 1) that can be applied to a range of behavioral problems (17, 18). We aimed to use the TDF to act as a step to identify factors relating to establishing and maintaining informational and management continuity and map them to behavioral domains so that further research can be directed to develop support interventions in Kerala.

**Table 1:** Demographic details.

**ID number**	**Age**	**Gender**	**Qualification**	**Job position**	**Clinical /administrative role**	**Years of experience**	**Experience with electronic health record**
HCP1	29	Male	Graduate	Doctor in PHC	Clinical	1 year and 5 months	No
HCP2	32	Female	Graduate	Staff nurse in PHC	Clinical	2 years	No
HCP3	28	Female	Post Graduate Diploma	Staff nurse in PHC	Clinical	3 years	No
HCP 4	34	Female	Post Graduate	Doctor in CHC	Clinical	12 years	No
HCP5	35	Female	Post Graduate	Doctor in FHC	Clinical	less than a year	Yes
HCP 6	50	Female	Post Graduate	Doctor in FHC	Clinical	20 years	Yes
HCP 7	38	Female	Post Graduate	Assistant Surgeon (FHC)	Administration	6 years	Yes
HCP 8	33	Female	Graduate	Staff nurse in FHC	Clinical	4 years	Yes
HCP 9	33	Female	Graduate	Staff nurse in PHC	Clinical	4 years	No
HCP 10	46	Female	Graduate	Assistant Surgeon (FHC)	Clinical	10 years	Yes
HCP 11	37	Male	Graduate	Doctor at PHC	Clinical	3 years	No
HCP 12	34	Male	Post Graduate	District Surveillance officer	Administration	less than a year	No
HCP 13	53	Female	Graduate	Medical Officer at Hospital	Clinical	20 years	No
HCP 14	40	Male	Post Graduate	Assistant Surgeon (FHC)	Clinical	8 years	Yes
HCP 15	42	Male	Post Graduate	Doctor at TQ Hospital	Clinical	6 years	No
HCP 16	37	Female	Post Graduate	District Surveillance officer	Administration	3 years	No
HCP 17	32	Male	Graduate	Doctor at FHC	Clinical	2 years	Yes

*PHC, primary health center; CHC, community health center; FHC, family health center; NCD, non-communicable disease*.

## Method

### Design

We re-analyzed data from a previous study using the TDF. Semi-structured interviews with 17 HCPs working in public health care facilities with experience of NCD programme (Supplementary Table S1) in Kerala were included. The data selected for this analysis were part of a qualitative study with patients, carers, and HCPs to explore their experiences with patient-held health records in Kerala. A paper-based patient-held health record (PHR) was developed as a clinically focused and primarily for HCPs to record clinical information. The PHR also contained additional information for patients, such as a generic diet plan for patients with diabetes and hypertension. However, owing to stock-outs, HCPs advised patients with diabetes and hypertension to buy themselves a notebook, which was used as PHR to be carried to public health facilities.

### Settings

We re-analyzed the data collected from HCPs working in public health settings in Kerala. The semi-structured interview data was collected from February to November 2020. In the pre-COVID phase, HCP data was collected at two FHCs in the Alappuzha district, Kerala, which is one of the first districts in which the NPCDCS was implemented in 2015. During COVID phase (March 2020 to November 2020), telephonic interviews were conducted with eligible HCPs working in public health facilities from other districts (Trivandrum, Ernakulam, Malappuram, and Wayanad).

### Sampling and Recruitment of HCPs

A purposive sampling (19) to ensure that HCPs with following experience were recruited; caring for patients with diabetes and hypertension working in public health facilities; work experience at FHCs with electronic health records under Mission Aardram. Convenience (20) and snowball sampling (21) were used to identify HCPs managing patients with diabetes and hypertension. Members of the research team contacted HCPs working in family health centers/primary health centers. Eligible and interested HCPs were given further information regarding study details by LJ.

### Data Collection

Data collection took place between February to November 2020. Semi-structured interviews were conducted face-to-face or telephonically based on HCPs' preferences. Face-to-face interviews were conducted in the doctors' room in FHC. The study investigator used a topic guide for the interviews (Supplementary Table S2). All interviews were done by LJ in Malayalam or English, or a mixture of both, based on interviewee preference. LJ was not previously known to HCPs. Interviews lasted 30–60 min and were audio-recorded. The audio recordings were transcribed to English and were checked by LTR against the audio, who was familiar with the clinical settings and Malayalam language.

The topic guide was developed and piloted before data collection to ensure the clarity of questions. The topic guide was informed by previous literature on handover communication and informational continuity and the working of health systems in Kerala. It included open questions regarding roles, responsibilities, and clinical practices of HCPs in managing care for patients with diabetes and hypertension, barriers to maintaining continuity of care, experiences with electronic health records and patient-held health records, and challenges in managing patients with diabetes and hypertension.

### Data Analysis

The data analysis followed the processes described by previous qualitative studies, which used the TDF (22–25). A deductive approach to content analysis (26) using the 14 TDF domains as the coding categories was done. Initially, LJ coded three transcripts at the domain level and developed a coding guideline (18) (Supplementary Table S3). The interview transcripts were coded line by line deductively against the TDF framework. SG and AL reviewed the coding guideline and accuracy of the coding of three transcripts. Two researchers (LJ and TL) independently coded the data manually in the next phase and met after coding five transcripts and then the remaining transcripts to discuss any variations. Any variations were discussed and resolved. Interview data could be placed in more than one domain.

After all interview transcripts were coded deductively within the domain level, Microsoft Excel was used to organize the coded data. LJ then generated data-driven statements relating to specific beliefs within each domain. Grouping statements by participants identified specific beliefs or sub-themes. The frequency of each belief (to represent the number of participants who mentioned the belief) was counted across all interviews. Domains were judged as likely to be relevant as barriers or facilitators if they fulfilled the following criteria; more than two HCPs mentioning the factor, high frequency of specific beliefs in a domain, presence of conflicting beliefs or strong beliefs that may influence maintaining informational continuity of care.

## Results

Of 17 HCPs interviewed, 13 were doctors, and four were nurses working in the public health facilities in Kerala. Respondents came from rural and urban public health care facilities with and without electronic health records implemented in their current workplace. All HCPs reported being responsible for care for patients with diabetes and hypertension in the outpatient settings (Table 1).

### Summary of Domains

Content analysis resulted in charting the interview data into all 14 domains of TDF (Table 2). The most frequently mapped domain was knowledge, and the least mapped domain was behavioral regulation. Thirty three specific beliefs were identified from the HCP interviews.

**Table 2:** Content analysis.

**Domains/categories**	**Factors affecting handover communication, informational and subsequent management continuity (sub-categories/specific beliefs)**	**Frequency count (out of 17)**
Knowledge	Knowledge regarding patient's past PHRs	17
	Awareness that patients may not carry records	15
	Awareness of NCD notebook in the public health system	17
	Awareness of patients returning to primary care centers for diabetes and hypertension treatment	8
Skills	Skills gained at the workplace	10
	Documenting skills	6
	Communicating with patients	3
	Uncertainty regarding skills in using electronic records	5
Social/professional role and identity	Role of the doctor in recording for informational continuity	13
	Nurses recognizing the role of a doctor in recording	3
Beliefs about capabilities	Low confidence in maintaining informational continuity	10
	Feeling that documentation needs to be prioritized over communication (conflicting priorities)	9
	Confidence in patients behavior returning to primary care centers for diabetes and hypertension treatment	8
	Confidence in maintaining continuity (Good follow-up care at primary care centers)	7
	Confidence in collecting information and recording in PHRs	6
Optimism	Optimistic about patients bringing documents	11
	Mixed feelings about maintaining informational continuity with electronic health records	8
	Ease of access information from electronic health record	7
Beliefs about consequences	Patient behaviors affecting informational and management continuity	16
	Patients' not bringing PHRs may result in information loss for us	10
	Not having PHR increases the potential for error	8
Reinforcement	Regular patients bring PHRs	7
Intentions	Taking measures to prevent information loss for us (HCPs)	15
Goals	Recording is based on HCP needs	12
Memory, attention, and decision processes	Lack of time for communication	7
	Interruption and difficulty in locating information	11
Environmental context and resources	Workload in the outpatient	17
	Potential advantage of electronic health record	9
	Workplace issues	4
Emotion	Treating patients who do not bring records is frustrating	3
Social influences	Encouraged to record in PHRs by senior colleagues	3
Behavioral regulation	Formats can help with recording	2
	Lack of supervision	1

Each mapped domain is summarized below with an illustrative quote. Additional quotes are presented in supplementary file (Supplementary Table S4).

### Knowledge

Most HCPs reported being aware of the booklet for patients with diabetes and hypertension issued as part of the NCD programme. They explained the reasons for booklets not being used in every health center due to lack of availability or stock-outs. Some HCPs reported that they did not receive any booklet for their center, and they had started asking patients to buy a notebook when they came to the health center for their diabetes and hypertension consultations. The reasons given by HCPs for maintaining a patient-held health record in the form of a notebook included the need for clinical information of patients, documenting medication prescriptions helps to track the patients to see if they have been regularly collecting medicines and to track the blood pressure or blood sugar values of patients.

*We give medicines, especially medicines, for patients with NCDs for 15 days, and then they have to come. You see there are no facility-based records. We do have some registers, but it will be very difficult to track who came, when, and such details. So if they bring the notebook, we can know. As a doctor, I think the notebook will give us a chance to monitor blood pressure and blood sugar. We are using a glucometer now as our lab technician is on leave, but still, we record it, and we can know if they had high BP previously. HCP 1*

All HCPs demonstrated awareness of patients' past PHRs and explained that patients might not carry PHRs to health care appointments.

*Yes. I have had experiences of patients' not bringing any records. It is in the past. In some situations, the patient may not be aware of the name of drugs or their own prior BP level. Few patients are there who come without knowing anything. Out of 50, only 1 or 2 are like that. Not 50; out of 100, one or two are like that. HCP 16*

### Skills

Most HCPs reported receiving some training on treatment guidelines for managing patients with diabetes and hypertension. HCPs reported not receiving any training for documentation in the PHRs from their current workplace. However, two doctors said that they received an orientation from the senior medical officer, who explained what types of records need to be maintained. Most doctors felt that they had picked up the content of documentation from their years of medical training and their workplace practices.

*No, these are the things (asking for PHRs/recording in them) we pick up in the workplace; there is no specific training as such for recording in PHR. HCP 11*

A few HCPs reported spending time communicating with the patient regarding managing their care when they are diagnosed with diabetes or hypertension. Both nurses and doctors shared the opinion that they communicate with patients to bring their NCD book when they come for renewing prescriptions.

*First of all, we inform the patients to bring a 200 page notebook. In the patient's book, address and code number will be there in front page. Inside pages, we mention the date and Doctor's write the prescribed medicines and to know are there any repetition or duplication of medicines. We tell them to bring it every time they come for buying medicines. HCP 3*

Most HCPs discussed that the training for electronic health records was important and useful. They reported that the focus was on setting up and having a mechanism for electronic health records to be incorporated into the consultations. However, a few HCPs discussed how some HCPs might be less skilled in entering the information in the electronic health record.

*Those with little computer knowledge or experience have difficulty in typing. It may be difficult for doctors who are older as they have followed a pattern and have built a system around themselves to work. They have their own traditional style, which they may not change. However, that can also be solved if we can get a data entry staff. HCP 15*

### Social/Professional Role and Identity

Healthcare providers, particularly doctors, saw themselves as responsible for documentation and maintaining records to prevent errors.

*If there is a notebook in our public health system, we doctors treat it as a record; we know we are in charge of recording the details of the consultation. HCP 6*

Participants who were doctors felt that they took extra efforts to maintain informational continuity by deciding to document in both electronic and patient-held records.

*We (doctors) continue to use the notebook. If we consult a patient this month, it may be some other doctor who deals with the patient on the next visit. There is still a chance to forget entering some details in the electronic health records. All (doctors) are new to this new system, and we may miss entering certain details. Most NCD (non-communicable disease) patients will bring the book (PHR), and we ask them to show it at consultations. Since we have only started the electronic ones (records), not all the past details may be fully entered into the system. Hence, it is good that we can refer to the notebook (PHR) so we maintain both currently.-HCP 5*

### Beliefs About Capabilities

In general, HCPs expressed confidence in documenting relevant information in PHRs and maintaining informational continuity. However, some HCPs reported difficulties in documenting in detail when the patient load is high in outpatient settings.

While most HCPs reported documenting in PHRs for informational continuity, some HCPs felt they had to prioritize documentation over communicating with patients in a busy outpatient setting.

*Sometimes in an OP, we will have a long queue, and then some emergency patients will arrive. I will have to go and attend; then, when I come back, I will be looking to finish off the OP patients. On top of that, we have many registers to maintain, so we will be writing in that than what we tell the patient. HCP 4*

Other factors that influenced HCPs' capabilities included confidence in other team members such as field workers (junior public health nurses and ASHAs) to follow-up patients with diabetes and hypertension to prevent dropping out from care.

*Generally, most people have interest to avail treatment for NCD from us. JPHN and ASHA workers have a good role in follow up care. Follow up is done correctly and continuously for the already existing patients. HCP 14*

HCPs also noted confidence in patient behavior to return to their health care facilities that HCPs perceived to be contributing to informational and management continuity of care.

*I have been practicing here for around 2 years. I know the existing NCD patients. So I will know if the patient is new or not. Most of our regular patients will come here and will bring their notebooks. If they have increased blood pressure or blood sugar in this visit, we may have to make judgements about changing the dose of medicines. HCP 16*

### Optimism

Most HCPs believed that patients would bring documents to consultations, particularly for diabetes or hypertension appointments. They also felt that if patient details are recorded well, there is potential for electronic health records to maintain informational continuity. However, some HCPs had mixed feelings about relying on electronic health records alone to maintain informational continuity. They pointed out that patients need to bring their unique identification card or phone number to retrieve patient information. Additional issues such as power failures or inadequate documentation were reported.

*Ideally, if everyone (HCPs) records the details properly in the case sheet in the electronic record, this will work. There will be information available for doctors in the next visit. But then we should be able to record, and the patient should bring their unique ID (identification card), or else it will not be useful. HCP 6*

### Beliefs About Consequences

Almost all HCPs spoke about the negative effects of not having adequate patient information. These included the potential for error, delays in arriving at proper treatment, and requesting additional follow-up. Some HCPs highlighted how having a PHR with recorded information is helpful for clinical decision-making.

*The advantages are that I will get to know the patient's condition for a longer time, and accordingly, I can change the medication increase or decrease, or if they are not responding to medication, then I can guide them to go to some MD specialist who knows better, who is better experienced and who can prescribe them better. HCP 13*

Most HCPs felt that they had experienced not having enough patient information during the consultation. Most HCPs reported that patients were familiar with carrying PHRs to diabetes and hypertension consultation, so they continue to write in them.

*We are still writing in the notebooks during consultation. Now we are typing the details in the electronic records and writing in the book so that if one fails, the other works. HCP 10*

### Reinforcement

Some HCPs held the enabling belief that regular patients brought PHRs to consultation and hence recorded in them as they felt satisfied in providing safe care.

*Most people who take treatment from us will continue to do so. They will be regular in bringing the papers, they will inform us if they have taken other treatment from outside, so it will be easier to write their records and treat them. HCP 13*

Some HCPs reported that they recorded key information from patients' past PHRs in the notebook for diabetes/hypertension to make their work later on easier.

*As doctors, I feel we will always be comfortable with patients who regularly come to get treatment from us because we know them, we have recorded the details we need. I think it is good and easy for doctors and patients. HCP 6*

### Intentions

Intentions are conscious decisions to perform a behavior. Most HCPs made a conscious decision to record in PHRs to prevent information loss. HCPs using electronic health records also reported deciding to record in PHRs and electronic records to maximize the possibility of information availability.

*I think that there is a possibility for power issues, or somehow the details were not recorded in the electronic record, so I will make it a point to write in the notebook. HCP 14*

*Ideally, we should not be using OP tickets now. But, we do it as the patient has to bring the unique health ID the next time they come. The card can be read and the details will be available for us. Now it is our headache when they miss it or they do not care about it. Again, the information is lost so we give them the OP tickets with prescriptions so that they will bring at least these. HCP 17*

*We are still writing in the notebooks during consultation. Now we are typing the details in the electronic records and writing in the book, so that if one fails the other works. HCP 10*

### Goals

Goals related to recording in the PHRs for informational and management continuity were described by HCPs as availability of relevant patient information for themselves. However, this task was not prioritized when the aim was to attend to all patients waiting in the outpatient clinic. Most HCPs referred to documenting in PHRs for them to have information on the patient's previous medicines to prevent medication errors.

Some HCPs inferred that there is an emphasis on recording information regarding medication for informational continuity rather than on test results.

*Yes, there is a focus on documenting the medicines majorly and maybe the BP readings. There are some deficiencies in recording from the doctors' side, but this is mainly due to the volume of work. They (other HCPs) have to cater to a large number of patients, around 150–200 in their OP. So the recording will be very much based on what they would need next time. HCP 12*

### Memory, Attention, and Decision Processes

Most HCPs believed that when there is a huge patient load in outpatient settings, they find it difficult to locate the necessary information from patients' multiple PHRs.

*But not everyone (patients) will carry records all time. There may be many papers also at times. Imagine having a long queue of patients outside your room, and then someone brings in many papers; it will take time to go through them to find what we need. It will interrupt the process of consultation. I think for new patients, we will have to sit through and check them, but with regular patients, it may be one or two here and there. HCP 1*

A few HCPs felt that they had to record crucial information such as the medication information (change the dose or medicine) and were found to have disregarded communicating test results to the patients.

*I have thought about it. Most of the time in the OP, I may not get time to teach them about diet and physical activities. We just check the blood values and blood pressure, and we change the dosage of medicine based on that. We may not even tell about the importance of blood values. This is what happens mostly during consultation time. HCP 5*

### Environmental Context and Resources

Almost all HCPs reported the high patient load in outpatient settings. HCPs discussed contextual factors that contribute to the potential loss of information and their own sub-optimal recording. One of the most discussed factors causing sub-optimal recording was the patient load during specific NCD days.

*There is like a huge number (of people) in primary care; the dire need is to cater to them and finish the consultations. HCP 12*

Another factor discussed by HCPs is the distraction caused by long queues and time pressures experienced by them to complete the consultations.

*The NCD days are hectic and noisy too. The pharmacy will also have a difficult time. We used to consult around 200 patients on NCD day. So there is not much to guess how much we can enter. It is not whether we know how to record, which I believe most of us can do but practically whether it is possible to write detailed notes. HCP 6*

A few HCPs felt that since there is no mechanism in place for auditing the patient-held records, which could possibly be a reason for sub-optimal recording.

### Social Influences

Some participants discussed the influence other people had on their recording and usage of PHR. A few HCPs described how senior doctors influenced their pattern of recording. Senior colleagues were perceived to provide information on content as well as the norms of recording behaviors in the health system.

*I have studied in a Government Medical College. During my training period, I have learned to document whenever we submit the records in different specialties from the seniors. We tend to do as they say to us. Even between departments, there will be some variations in what we record. We will usually follow what has been done before. HCP 12*

### Emotion

Only a few HCPs expressed their difficulty in providing care for patients who do not bring their records, and this leaves them frustrated as they will have to insist the patients go back and take their records or refuse medications.

*But some patients may come without a prescription and tell three tablets for blood pressure, four tablets for some other problem, three yellow tablets, or round tablets. They are the more problematic persons for us. It becomes difficult then, they have medicines for BP, but we do not know which one, and we may have to insist them to go and bring the papers. For them, it is their medicine; they probably do not realize that many tablets are round. HCP 6, doctor in FHC*

### Behavioral Regulation

Only a few HCPs suggested having formats or templates for recording relevant information can help with easier documentation.

*I think having some template will be useful. See most of the NCD patients will have some standard medicines, so having them printed out in the booklet and in the EHR will definitely help with recording. HCP 10*

One HCP highlighted the lack of supervisory checks for PHRs may be a potential reason for sub-optimal documentation.

### Barriers and Enablers Identified Within Relevant Domains

**Figure 1:**
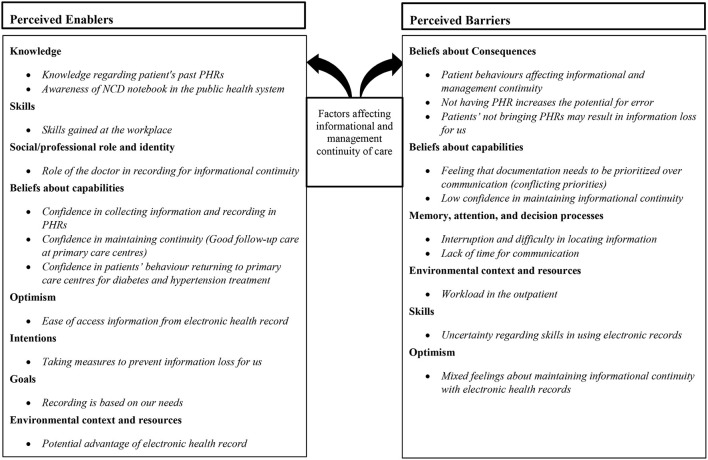
Factors affecting informational and management continuity of care.

The domains relevant for factors influencing continuity of care are summarized in Figure 1. The most frequently perceived enablers for recording in PHRs and maintaining continuity of care treatment fell into the following domains: “knowledge”, “skills”, “social or professional role and identity”, “beliefs about capabilities”, “intentions”, “goals” and “optimism”. All participants indicated that patient behaviors in carrying records act as a barrier to informational continuity (“beliefs about consequences”). The most frequently perceived barriers for recording in PHRs and maintaining informational continuity fell into the following domains “memory, attention and decision processes” “environmental context and resources” domain; these were in relation to limited resources or capacity and the challenges presented by the health system leading to perceived barriers to capabilities and skills.

## Discussion

This study aimed to use TDF to identify the factors influencing the establishment and maintenance of informational and management continuity for patients with diabetes and hypertension. This was done using capturing perspectives of health care providers working in public (government) health facilities providing care for patients with diabetes and hypertension in Kerala. The data were mapped into the 14 domains of the TDF. We identified most frequently perceived enablers for recording in PHRs and maintaining continuity of care treatment fell into the domains of knowledge, skills and professional role and identity. Whilst some other domains such as beliefs about capabilities, intentions, goals and optimism had some specific beliefs which suggests being a facilitator for informational continuity. The most frequently perceived barriers were in the mostly in two domains. The domains were “memory, attention and decision processes”, “environmental context and resource” domains.

### Facilitators

This study showed that knowledge and skill are interlinked for maintaining informational continuity and subsequent management continuity using PHRs. For example, knowledge of the availability of PHRs for patients with diabetes and hypertension prompted HCPs to record in them. HCPs were aware of the working of the public health system and the need for maintaining PHRs due to the absence of facility-based records or perceived difficulties in the retrieval of information from electronic health records. Furthermore, doctors identified their role in documenting in PHRs and maintaining informational continuity for themselves. Even though the TDF does not discuss the relationship between domains, this study suggests a link between the professional identity and confidence of doctors in recording in PHRs. Similar findings, which linked knowledge, and skills domains have been reported for prescribing behaviors among trainee doctors (27).

### Barriers

Most of the barriers to continuity of care were attributed to beliefs about consequences and capabilities due to patients' behavior. One of the reasons for this attribution to patients' behavior as HCPs are reliant on patients/carers to bring their past records, communicate their health information to maintain informational continuity of care. Additionally, this finding highlights whether it is possible to rely on clinicians alone to critically evaluate their behaviors, which may threaten the continuity of care. Similar findings have been reported when using the TDF for identifying barriers and facilitators to prescribing behaviors. Studies have reported that clinicians may attribute the deviations in clinical behaviors to other external factors such as environmental factors or issues with lack of resources (23, 27). The above suggests that there may be a need for using cultural models that may explain the variations in HCPs' clinical practices. Although HCPs did not identify a lack of training for documentation in PHRs that may be a barrier to adequate documentation in the PHRs. Additionally, a few beliefs may contribute to being barriers to maintaining informational continuity. HCPs believed that patients who have come to seek treatment in public health settings would continue to do so. However prior studies from India have shown that patients have a tendency to “shopping” for care across HCPs (7, 11, 12, 28). Along with this, the overall goal for recording in PHRs for HCPs in this study is focussed on having information for themselves. These beliefs could be a possible reason for not communicating the importance of PHRs for all HCPs to patients and carers.

The findings from the study indicate domains such as belief about consequences, reinforcement and optimism have specific beliefs associated with anticipated and experienced patient behavior such as bringing records or communicating with HCPs. In the domain reinforcement, HCPs reported that patients who regularly come to their health facility for appointments brought previous PHRs. Previous studies have suggested that informational continuity of care is built over time (3, 4). For informational continuity to be achieved patients/carers also need communicate their symptoms, and other relevant information to HCPs that could be recorded. HCPs within this study have reported multiple factors that may contribute to be a barrier or an enabler. Some factors contribute to a number of domains. For example, patient behavior of bringing multiple records impacted on belief about consequences, belief about capabilities and memory attention and decision-making.

### Role of Electronic Health Records and NCD Notebook in Continuity of Care

Overall HCPs had mixed opinions regarding electronic health records and their role in maintaining informational continuity. Some HCPs felt that if electronic health records are being used widely in the health system and regularly documented well, they have a potential for improving informational availability. Only one HCP felt that using PHRs along with electronic health records would add to documentation burden. Most HCPs felt since they are familiar with recording in PHRs such as NCD notebooks, having both forms of documentation would ensure a better chance of maintaining continuity of care. HCPs regarded patient held NCD notebooks as records and reported on using them for the information recorded. These findings are similar to other LMICs, which have been using paper as documentation interface (29, 30). Further research in having both paper-based PHRs and electronic health records for improving quality of care is needed in these settings (31). Further research is needed to map the intervention content for any behavior change intervention for HCPs that should include basic training sessions (32).

### Strengths and Limitations

This study used qualitative methods to explore HCPs' practices of recording in PHRs regarding the management of patients with diabetes and hypertension and to use the TDF to explore factors affecting informational and subsequent management continuity of care. Incorporating the TDF ensured the data was coded and analyzed using a recognized framework that can help in future intervention development processes. However, further research should also incorporate the interactive nature of the communication process and the development of informational continuity. This will give insights into healthcare providers' and patients' power dynamics and relationships.

There are a few limitations of this study. The interview guide was not developed using the TDF framework and hence could have overlooked some domains, which could have been potentially relevant. For example; only a few HCPs mentioned influences of senior doctors on their recording behavior. This social influence of senior doctors and other health care workers have been previously reported with blood transfusion practices (25), prescribing behaviors of trainee doctors (27) and nurses (23). However, not using the TDF to develop the interview guide gave the advantage of capturing contextual information that could potentially explain interviewees' behaviors.

## Conclusion

Using theory, we identified a range of determinants for HCPs in recording in PHRs and maintaining continuity of care. We identified the high workload, influence of patient behaviors and interruptions in outpatient settings that act as barriers to enact the behaviors. This led HCPs to prioritize recording sub-optimally. We offer new insights into the intentions and goals of HCPs when using PHRs; the recording in PHRs is to maintain informational continuity for HCPs. Therefore, comprehensive recording in medical records, which can enable informational continuity for all future HCPs should be one areas of targeted intervention. Next, communicating with patients/carers the importance of carrying documented medical information, and use of PHRs for patients or carers to interact with all HCPs should be targeted for intervention development. This study suggests that knowledge, skill and professional identities are associated with positive HCPs' behaviors relating to maintaining continuity of care. Further research is needed to map the intervention content for any behavior change intervention for HCPs and it should consider the existence of electronic as well as paper-based PHRs.

## Data Availability Statement

The raw data supporting the conclusions of this article will be made available by the authors, without undue reservation.

## Ethics Statement

The studies involving human participants were reviewed and approved by Center for Chronic Disease Control, New Delhi, India [CCDC_IEC_05_2019] and the University of Birmingham, UK [ERN_18-1933]. The patients/participants provided their written informed consent to participate in this study.

## Author Contributions

LJ designed the study with the support of SG, SM-H, AL, and JP. SG, SM-H, AL, and JP supervised the project. TL worked with LJ as second qualitative coder. LJ prepared the first draft. All authors gave critical feedback on the manuscript, contributed to the article and approved the submitted version.

## Funding

LJ was supported by the University of Birmingham Global Challenges Ph.D. scholarship.

## Conflict of Interest

The authors declare that the research was conducted in the absence of any commercial or financial relationships that could be construed as a potential conflict of interest.

## Publisher's Note

All claims expressed in this article are solely those of the authors and do not necessarily represent those of their affiliated organizations, or those of the publisher, the editors and the reviewers. Any product that may be evaluated in this article, or claim that may be made by its manufacturer, is not guaranteed or endorsed by the publisher.
