# Supplementary material for: Exploring Factors Affecting Health Care Providers' Behaviors for Maintaining Continuity of Care in Kerala, India; A Qualitative Analysis Using the Theoretical Domains Framework
Source: Front Public Health. 2022 Jul 8;10:891103. doi: 10.3389/fpubh.2022.891103 (PMC9304901; doi:10.3389/fpubh.2022.891103)
Supplement: Supplementary file 1 [file Table_1.DOCX]

**Table S1 Overview of non-communicable disease programme**

| **India**  The Government of India launched the National Programme for Prevention and Control of Cancer, Diabetes, Cardiovascular Diseases and Stroke (NPCDCS) in 2008 and later merged into the National Health Mission (NHM) in 2013 to integrate the NCD-related programme activities such as health promotion, early diagnosis, treatment, and referral. The federal structure of the Indian health system mandates shared responsibility of health by states and central government. The national policies set the directions and allocate funds for the programmes, while the states are responsible for establishing and maintaining the public health infrastructure and health care activities. |
| --- |
| **Kerala**  In Kerala, NCD programme activities included early detection, treatment, and referral facilities at the primary and secondary public health facilities. Public health centres (PHC) provided care at the primary level and community health centres and district hospitals at the secondary level. Patients with diabetes and hypertension could access treatment and follow-up care at PHCs. A paper-based patient-held health record (PHR) was developed as a clinically focused and primarily for HCPs to record clinical information. The PHR also contained additional information for patients, such as a generic diet plan for patients with diabetes and hypertension. However, owing to stock-outs, HCPs advised patients with diabetes and hypertension to buy themselves a notebook, which was used as PHR to be carried to public health facilities. Along with this, both national and state governments have pledged to deliver comprehensive primary health care services to the entire population. The government of Kerala has begun a programme to upgrade and transform all primary health centres in states as part of the Mission Aardram programme and renamed these facilities as Family Health Centres (FHC). These facilities focus on upgrading the infrastructure, establishing electronic health records, and providing more doctors and staff in closer collaboration with elected rural governments (panchayat) members. |

**Table S2 Health care provider interview topic guide**

| Healthcare provider topic guide   - Could you please walk me through your typical day like in FHC (or PHC/CHC/hospital)? - Could you please tell me about how you manage patients with diabetes and hypertension in your centre? - Could you please tell me if people mention access as an issue for their care? - Could you tell me in your opinion issues relating to management of patients with diabetes and hypertension in your centre? - Have you got a chance to look at patient’s lab reports? - Could you please tell me what kind of notes patients bring to consultation? - How do you currently manage any records brought by patients to FHC?   *Could you please tell me what do you normally do with other OP sheets/ reports brought by patients?*   - What written documentation do you provide the patients with diabetes and hypertension and other NCD patients?   *Any advice regarding how to use these documents for themselves, other HCPs*   - In your opinion, do patients take this book/papers to other providers? - What do you think about not having documented patient’s health information from other providers?   *One or more examples when you needed the papers from other HCPs, how did you manage without past information?*   - In your experience and opinion can patients explain what has happened in the previous doctor visit? - In your experience how do you provide information on caring for themselves to patients/carers?   *Verbal information, booklet/recorded information*   - Could you please describe your experience with recording in electronic health records?   *What was the best thing about it? What was the most difficult? How does it affect information retrieval and handover communication? What are your thoughts on electronic health records current implementation?* |
| --- |

**Table S3 Coding Framework**

| Domain (definition) | Constructs | Description relevant to the study |
| --- | --- | --- |
| 1. Knowledge (An awareness of the existence of something) | Knowledge (including knowledge of condition/scientific rationale) Procedural knowledge Knowledge of task environment | Awareness of the availability of PHRs for patients with diabetes/hypertension. Awareness of using PHRs for informational continuity for providers and patients. Procedural knowledge on using PHR for recording health care given and health care information regarding patient. Awareness of health education available in the PHR. |
| 2. Skills (An ability or proficiency acquired through practice) | Skills Skills development Competence Ability Interpersonal skills Practice Skill assessment | Recording skill-without errors  Requesting PHR  Using PHRs to explain own care management |
| 3. Social/professional role and identity (A coherent set of behaviours and displayed personal qualities of an individual in a social or work setting) | Professional identity Professional role Social identity Identity Professional boundaries Professional confidence Group identity Leadership Organisational commitment | Doctors stating recording (maintain records for informational continuity) as their responsibility or as a recognised practice.  Other statements reflecting the role of doctors/nurses in communicating the care plan with patients/family, preventing medication errors, being efficient with time and resources and patient safety.  Role of other health care providers such as nurses or pharmacists in recording, preventing errors, communicating with patients/families. |
| 4. Beliefs about capabilities (Acceptance of the truth, reality or validity about an ability, talent or facility that a person can put to constructive use) | Self-confidence Perceived competence Self-efficacy Perceived behavioural control Beliefs Self-esteem Empowerment Professional confidence | How HCPs are able to ask, record and maintain informational continuity using PHRs. |
| 5. Optimism (The confidence that things will happen for the best or that desired goals will be attained) | Optimism Pessimism Unrealistic optimism Identity | Statements reflecting how the use of PHRs can improve informational continuity for HCPs, how electronic health records can contribute to informational continuity. |
| 6. Beliefs about Consequences (Acceptance of the truth, reality, or validity about outcomes of a behaviour in a given situation) | Beliefs Outcome expectancies Characteristics of outcome expectancies Anticipated regret Consequents | Perceptions about outcomes, advantages and disadvantages of using PHR for informational continuity for HCPs and patients. |
| 7. Reinforcement (Increasing the probability of a response by arranging a dependent relationship, or contingency, between the response and a given stimulus) | Rewards (proximal/distal, valued/not valued, probable/improbable) Incentives Punishment Consequents Reinforcement Contingencies Sanctions | Increasing, or decreasing, the probability of using PHRs for patients with diabetes/hypertension due to a relationship between consequents and using PHR; constructs of associative learning (e.g. motivated to record in PHR due to repeated positive experiences). |
| 8. Intentions (A conscious decision to perform a behaviour or a resolve to act in a certain way) | Stability of intentions Stages of change model Trans-theoretical model and stages of change | A conscious decision to perform a behaviour (requesting PHR from patients with diabetes/hypertension, recording in PHRs and explaining own health care management at home to patients using PHR). |
| 9. Goals (Mental representations of outcomes or end states that an individual wants to achieve) | Goals (distal/proximal) Goal priority Goal/target setting Goals (autonomous/controlled) Action planning Implementation intention | Priorities, importance or commitment to providing informational continuity using PHRs to other providers, discussion of informational continuity and own care management for patients with diabetes and hypertension. |
| 10. Memory, attention and decision processes (The ability to retain information, focus selectively on aspects of the environment and choose between two or more alternatives) | Memory Attention Attention control Decision making Cognitive overload/tiredness | The processes involved, and the factors taken into account (e.g. patient circumstances), when making the decision about recording (eliciting previous medical history and reminding patients regarding health care activities at home. |
| 11. Environmental context and resources (Any circumstance of a person’s situation or environment that discourages or encourages the development of skills and abilities, independence, social competence and adaptive behaviour) | Environmental stressors Resources/material resources Organisational culture/climate Salient events/critical incidents Person × environment interaction Barriers and facilitators | Any circumstance of doctors’ situation or environment that encourages or discourages providing appropriate care for patients with diabetes/hypertension using PHR (including people and organisational factors e.g. resources, client circumstances). |
| 12. Social influences (Those interpersonal processes that can cause individuals to change their thoughts, feelings, or behaviours) | Social pressure Social norms Group conformity Social comparisons Group norms Social support Power Intergroup conflict Alienation Group identity Modelling | External pressure from other people that influence the decision to record/use PHR e.g. encouragement from patients or colleagues, request from patients/caregivers for documented medical information regarding their condition |
| 13. Emotion (A complex reaction pattern, involving experiential, behavioural, and physiological elements, by which the individual attempts to deal with a personally significant matter or event) | Fear Anxiety Affect Stress Depression Positive/negative affect Burn-out | Emotions are feelings about providing care for patients with diabetes/hypertension and using PHRs (e.g. fear, anxiety, stress). |
| 14. Behavioural regulation (Anything aimed at managing or changing objectively observed or measured actions) | Self-monitoring Breaking habit Action planning | Behavioural regulation refers to ways of doing things, at an individual or environmental level, that relate to pursuing and achieving desired goals (e.g. using PHR for patients with diabetes and hypertension to maintain informational continuity). |

**Table S4 Additional illustrative quotes based on domains**

| **Domain** | **Sub-themes** | **Interview data** |
| --- | --- | --- |
| Knowledge | Knowledge regarding patient's past PHRs | They will bring some records. Some patients pin the whole OP sheets as a bulk. HCP 3 |
|  |  | Yes, patients bring old medical records. HCP 17 |
|  | Awareness that patients may not carry records | Usually patients will come with OP sheets but if they have miss placed or lost it that, they will come for another OP sheet. HCP 16 |
|  |  | In some situations, patients may not bring any records nor do they know their medicines name or past blood pressure levels. HCP 15 |
|  | Awareness of NCD notebook in the public health system | We have an NCD book for people with diabetes/hypertension and they bring the book with them for each visit. HCP 14 |
|  |  | Now that eHealth is implemented, there is a lot less paperwork. Because once we give the token to the patient at the registration, the same details (demographic) will be available until pharmacy. Although we cannot follow that always. In the case of NCD patients, they will have a notebook or for older citizens, they have another one (booklet). For them we still use them. We will write the token number inside the book so that the doctor can write it in the book. HCP 8 |
|  | Awareness of patients returning to primary care centres for diabetes and hypertension treatment | Most like around 80% of NCD patients come to FHC for buying their repeat medicines. Others come if they experience any difficult or they feel like they need to check their blood values (such as Fasting Blood Glucose). HCP 10 |
| Skills | Skills gained at workplace | No these are the things (asking for PHRs/recording in them) we pick up in the work place, there is no specific training as such for recording in PHR. HCP 11 |
|  |  | No training as such we have seniors do the things and we do the same thing. HCP 13 |
|  |  | We used to consult around 200 patients on NCD day. Therefore, there is not much to guess how much we can enter. It is not whether we know how to record, which I believe most of us can do but practically whether it is possible write detailed notes. HCP 6 |
|  | Communicating with patients | When a patient is initially diagnosed with diabetes, I tell them to take their medicines regularly, control their diet and walk for at least 30 minutes. HCP 4 |
|  |  | I will tell the patients like I find that you are improving with medicine as I see all the records so I let them know I will reduce the dose of medicine. HCP 13 |
|  |  | In case of old age people, they may have their book or else we suggest the patients to bring any type of book. If we simply issue a number, they may miss it to bring next time. So, we usually advise them to buy a book and we enter the identification number on it. We tell them to bring it to health centre when they come to buy medicines for diabetes or hypertension. HCP 8 |
|  |  | During their (patients’) first visit itself, we tell them to bring a notebook, Aadhar, ration card and phone number. We record their details and tell them to bring notebook the next time. Later they bring the notebook for every subsequent visit. In case, if they have any difficulties we advise them to come after 1 week. HCP 9 |
|  | Training for electronic records | They have trained us. Initially Dr X who taught us regarding the electronic system. Later department posted some candidates to provide such training. There were 2 permanent staff for more than 2 weeks with us, up to last December to January. However, during lockdown they have withdrawn one candidate. HCP 8 |
|  |  | Usually patients with NCD have continuous medications. There is no option in the electronic records to repeat them. Every time we need to type it, it is difficult but I am sure with practice it should be better. HCP 10 |
|  | Uncertainty regarding skills in using electronic records | Those with little computer knowledge or experience have difficulty in typing. It may be difficult for doctors who are older as they have followed a pattern and have built a system around themselves to work. They have their own traditional style, which they may not change. However, that can also be solved if we can get a data entry staff. HCP 15 |
|  |  | Practically what happens is patients take one registration and get the card and unique ID. But they will not bring it the next time. So what is the point in having the electronic record, if each time we are having to issue temporary IDs, then there is no information being entered in the system, then how will we get the information next time? HCP 6 |
| Social/professional role and identity | The role of the doctor in recording for informational continuity | If there is a notebook in our public health system, we doctors treat it as a record; we know we are in-charge of recording the details of the consultation. –HCP 6 |
|  |  | We (doctors) continue to use the notebook. If we consult a patient this month, it may be some other doctor who deals with the patient on the next visit. There is still a chance to forget entering some details in the electronic health records. All (doctors) are new to this new system and we may miss entering certain details. Most NCD patients will bring the book and we ask them to show it at consultations. Since we have started the electronic ones, not all the past details may be fully entered into the system. Hence, it is good that we can refer to the notebook so we maintain both currently.-HCP 5 |
|  | Nurses' recognising doctors' role in recording in PHR | Doctors usually write the medicines and the blood reports. We check BP and GRBS every month. In addition, HbA1c every three months. Doctors write these details in patient’s notebook and we will copy them in the treatment card kept at the PHC. If there is any change in medicines doctors will write, it and they will also add the review date in the treatment card. HCP 2 |
|  |  | In the patient’s book, address and code number will be there in front page. Inside pages, we mention the date and Doctor’s write the prescribed medicines and check for any repetition or duplication of medicines. HCP 3 |
| Beliefs about capabilities | Confidence of collecting information and recording in PHRs | So if it is a new patient, I will ask for detailed past medical history and document it in the notebook and initiate treatment based on his blood pressure/sugar values. HCP 15 |
|  |  | It is not whether we know how to record, which I believe most of us can do but practically whether it is possible write detailed notes. HCP 6 |
|  | Feeling that documentation needs to be prioritized over communication (conflicting priorities) | Sometimes in an OP, we will have a long queue and then some emergency patient will arrive. I will have to go and attend, and then when I come back, I will be looking to finish off the OP patients. On top of that, we have many registers to maintain, so we will be writing in that than what we tell the patient. HCP 2 |
|  | Minimal information recorded | Usually, we write the basic patient details in the OP ticket, but if the patient does not bring it, then it is not of much use. HCP 10 |
|  | Low confidence in maintaining informational continuity | When there is a lot of patients in the OP, the documentation will get affected. I will be focusing on writing the medicines at least, or if they are same from last time, will document “repeat all”. HCP 10 |
|  | Confidence in maintaining continuity (Good follow-up care at primary care centres) | Most people who have taken treatment from us will avail treatment for NCDs from us. Our Junior Public Health Nurses (JPHN) and Accredited Social Health Activist (ASHA)s play an important role in following people up and ensuring that they continue their treatment here (PHC). HCP 15 |
|  | Confidence in patients behaviour returning to primary care centres for diabetes and hypertension treatment | I have been practising here for around 2 years. I know the existing NCD patients. So I will know if the patient is new or not. Most of our regular patients will come here and will bring their notebook. If they have increased blood pressure or blood sugar in this visit we may have to make judgements about changing the dose of medicines. HCP 15 |
| Optimism | Ease of access information from electronic health record | After using electronic health records, it is easy to get patients’ history. Particularly we can know the medicines they take. Once we enter the details doctors can view the details in the subsequent visits. HCP 10 |
|  | Mixed feelings about maintaining informational continuity with electronic health records | Ideally if everyone records the details properly in the case sheet in the electronic record this will work. There will be information available for doctors in the next visit. Then we should be able to record and the patient should bring their unique ID (identification card), or else it will not be useful. HCP 6 |
|  | Optimistic about patients bringing documents | Patients bring their notebooks or any documents they get from other doctors. We consider the documents, which patients bring as authentic. Besides these are the best available option or the only option. HCP 13 |
|  |  | They always carry the book to hospital on each visit. HCP 3 |
| Beliefs about Consequences | Patient behaviours affecting informational and management continuity | I feel from patients’ perspective they are getting free treatment and medicines and follow up from PHCs. Sometimes there will be an interruption to treatment. In case a pregnant mother was taking, medicines from this PHC might go to her house for delivery and then there will be definitely issues with information being transferred. They may eventually reach back the system for treatment but not sure when they will be back, or whether they continued their treatment, for all these information we have to depend on the patients. HCP 15 |
|  |  | It is not that we do not get medical information from patients. Sometimes the information may not be clear. This where I have found it most difficult. They will verbally inform us they are hypertensive and the BP values may suggest that. Then they will say I am taking a 500 mg tablet. In such cases, I ask them to get the papers from home. HCP 6 |
|  | Not having PHR increases potential for error | But some patients may come without a prescription and tell three tablets for blood pressure, four tablets for some other problem, three yellow tablets, or round tablets. They are the more problematic persons for us. It becomes difficult then, they are having medicines for BP but we don’t know which one and we may have to insist them to go and bring the papers. For them, it is their medicine, they probably don’t realise that many tablets are round. HCP 6, doctor in FHC |
|  |  | Once a COPD (Chronic Obstructive Pulmonary Disease) patient came to FHC, we were not aware that he had this condition (COPD). The patient did not mention it nor did have any records with him. We (the doctor) prescribed him a particular antihypertensive. Later we understood he is a COPD patient. Then he came onto the next visit, with the prescription of a pulmonologist and we changed the anti-hypertensive for him. So now, the whole treatment was affected. HCP 5 |
|  |  | In case of diabetic patients, we refer them to GH if they have any renal issues. We get their creatinine tested and refer them. However, when they come back to us, they insist on taking their previous medicines. When they do not bring back the notes, they got at the hospital it is difficult for us to know what has happened at the hospital. HCP 10 |
| Reinforcement | Regular patients bring PHRs | Most people who take treatment from us will continue to do so. They will be regular in bringing the papers, they will inform us if they have taken other treatment from outside, so it will be easier to write their records and treat them. HCP 13 |
|  |  | Mostly, our regular patients inform us they went for treatment in other hospital in the previous month and will bring the records to show the doctor. HCP 3 |
|  |  | Once a COPD (Chronic Obstructive Pulmonary Disease) patient came to FHC, we were not aware that he had this condition (COPD). The patient did not mention it nor did have any records with him. We (the doctor) prescribed him a particular antihypertensive. Later we understood he is a COPD patient. Then he came onto the next visit, with the prescription of a pulmonologist and we changed the anti-hypertensive for him. So now, the whole treatment was affected. HCP 5 |
|  |  | In case of diabetic patients, we refer them to GH if they have any renal issues. We get their creatinine tested and refer them. However, when they come back to us, they insist on taking their previous medicines. When they do not bring back the notes, they got at the hospital it is difficult for us to know what has happened at the hospital. HCP 10 |
| Goals | Recording is based on our needs | Yes, there is a focus on documenting majorly the medicines and may be the BP readings. There are some deficiencies in recording from the doctors' side but this is mainly due to the volume of work. They have to cater to a large number of patients around 150-200 in their OP. So the recording will be very much based on what they would need next time. HCP 12 |
|  |  | The documenting becomes very much what we need the next time. Most patients who take treatment from here will come back here. HCP 14 |
| Memory, attention and decision processes | Lack of time for communication | I have thought about it. Most of the time in the OP, I may not get time to teach them about diet and physical activities. We just check the blood values and blood pressure and we change the dosage of medicine based on that. We may not even tell about the importance of the blood values. This is what happens mostly during consultation time. HCP 5 |
|  |  | I try to make it a point to communicate with new patients regarding how to manage but sometimes there is a rush and I will not have enough time with patients. HCP 1 |
|  | Interruption and difficulty in locating information | But not everyone (patients) will carry records all time. There may be many papers also at times. Imagine having a long queue of patients outside your room and then someone brings in many papers; it will take time to go through them to find what we need. It will interrupt the process of consultation. I think for new patients we will have to sit through and check them, but with regular patients it maybe one or two here and there. HCP 1 |
|  |  | They (patients) have documents, which contain details like medicines, investigations, RBS value and BP value alone. No doctors will get enough time to write all stories during busy OP’s. Some time they may not write diagnosis also. If they consult in a big private hospital, they may enter the detailed diagnosis but in small PHC’s diagnosis is like hypertension or type 2 diabetes. There will not be any detailed diagnosis or family medical history because they will not get time. There is no point in discussing that. In a single NCD day, there may be around 150-200 patients at a time. We used to consult 200 to 220 patients that is one doctor in a single day. Therefore, there is not much to guess how many records can we enter? It is not about whether they know which they know but practically will not be able to write detailed notes. HCP 6 |
|  |  | After a referral to GH (general hospital), three things can happen. Mostly people will return to us with the OP ticket or sheet; others come and verbally report what happened at the consultation at GH. The last category never will come back. HCP 10 |
| Environmental context and resources | Workload in the outpatient | Sure, we do enter some family history but can’t do it all time because of heavy workload and rush in the morning and evening O.P is peak time and almost 250 patient daily. HCP 8 |
|  |  | There is like a huge number (of people) in primary care, the dire need is to cater to them and finish the consultations.  HCP 12 |
|  |  | But not everyone (patients) will carry records all time. There may be many papers also at times. Imagine having a long queue of patients outside your room and then someone brings in many papers, it will take time to go through them to find what we need. It will interrupt the process of consultation. I think for new patients we will have to sit through and check them, but with regular patients it maybe one or two here and there. HCP 1 |
|  |  | They (patients) have documents, which contain details like medicines, investigations, RBS value and BP value alone. No doctors will get enough time to write all stories during busy OP’s. Some time they may not write diagnosis also. If they consult in a big private hospital, they may enter the detailed diagnosis but in small PHC’s diagnosis is like hypertension or type 2 diabetes. There will not be any detailed diagnosis or family medical history because they will not get time. There is no point in discussing that. In a single NCD day, there may be around 150-200 patients at a time. We used to consult 200 to 220 patients that is one doctor in a single day. Therefore, there is not much to guess how many records can we enter. It is not about whether they know, which they know but practically will not be able to write detailed notes. HCP 6 |
|  |  | After a referral to GH (general hospital), three things can happen. Mostly people will return to us with the OP ticket or sheet; others come and verbally report what happened at the consultation at GH. The last category never will come back. HCP 10 |
|  | Potential advantage of electronic health record | It is easy to get patients history especially patient’s medical history. We get to know what medications they have taken. Usually, we write in the OP tickets and most of the patients may not bring it. But in the E-system, if we enter all details at first, doctors can view the same during the subsequent visits. By looking at records it easy for us to understand the patients. We write bio data and diagnosis clearly which helps everyone to understand and do tally easily. HCP 10 |
|  |  | Now that eHealth is implemented, there is a lot less paperwork. Because once we give the token to the patient at the registration, the same details (demographic) will be available until pharmacy. HCP 8 |
|  |  | Since eHealth is not fully implemented and functional in the health system. It will be difficult to say whether there is any need for paper based records. If everything works according to plan, of course it is a good thing for us. HCP 6 |
|  | Workplace issues (lack of supervision, hierarchical issues) | Then these issues as some doctors who work hard will continue their work but some people are there do not work at all. Therefore, like some sort of hierarchy, which makes some work more, and they may have difficulty to give more care to patients. HCP 12 |
| Emotion | Treating patients who do not bring records is frustrating | People who do not bring papers and then say they take medicines for sugar or pressure then that becomes more difficult. If it was a new patient, I would have started them on something but now they have already been on medicines but we don’t know what it is, that’s really disappointing. HCP 4 |
| Social influence | Encouraged to record in PHRs by senior colleagues | I used to ask doctors who joined at my PHC to record well. Although there is no stipulated checks for documentation, I used to guide them if they had any issues. We are more focused on maintaining our registers at PHC, which will be audited. HCP 16 |
| Behavioural regulation | Formats can help with recording | Currently, there is no such option in the E system. Templates of each patient show different combinations of drugs. Patients come every two week for medicines. If the system shows all repeated medicines or displays ‘continuously taking’ for the patients who come for frequent check-ups, which would be very useful. HCP 10 |
|  | Lack of supervision | Then, there is no method anywhere, to improve the staffs those who work less. Even if they do not document well, there is no checks or penalty. HCP 12 |
